# How Do Drugs Affect the Skeleton? Implications for Forensic Anthropology

**DOI:** 10.3390/biology11040524

**Published:** 2022-03-29

**Authors:** Nicholas Márquez-Grant, Elisa Baldini, Victoria Jeynes, Lucie Biehler-Gomez, Layla Aoukhiyad, Nicholas V. Passalacqua, Gaia Giordano, Domenico Di Candia, Cristina Cattaneo

**Affiliations:** 1 Cranfield Forensic Institute, Cranfield University, Bedford MK43 0AL, UK; elisa.baldini.uk@gmail.com; 2 Urgent Primary Care, Cwm Taf Morgannwg University Health Board, Cardiff CF14 4XW, UK; v.jeynes@outlook.com; 3 Department of Biomedical Sciences for Health, Università degli Studi di Milano, 20122 Milano, Italy; lucie.biehler@unimi.it (L.B.-G.); gaia.giordano1@gmail.com (G.G.); domenico.dicandia@unimi.it (D.D.C.); cristina.cattaneo@unimi.it (C.C.); 4 Pharmacist, Servei de Salut de les Illes Balears, 07003 Palma, Spain; lilitfar@yahoo.es; 5 Anthropology and Sociology Department, Western Carolina University, Cullowhee, NC 28723, USA; passala5@gmail.com

**Keywords:** forensic anthropology, medication, drugs of abuse, biological profile, human identification

## Abstract

**Simple Summary:**

Forensic anthropologists analyze human remains to assist in the identification of the deceased, predominantly by assessing age-at-death, sex, stature, ancestry and any unique identifying features. Whilst methods have been established to create this biological profile of the skeleton, these may be influenced by a number of factors. This paper, for the first time, provides an overview from a reading of the clinical and pharmacological literature to explore whether the intake of drugs can affect the skeleton and whether these may have implications for forensic anthropology casework. In effect, drugs such as tobacco, heroin, and prescription medications can alter bone mineral density, can increase the risk of fractures, destroy bone and changes to the dentition. By considering how drugs can affect the skeleton, forensic anthropologists can be aware of this when attempting to identify the deceased.

**Abstract:**

Forensic anthropologists rely on a number of parameters when analyzing human skeletal remains to assist in the identification of the deceased, predominantly age-at-death, sex, stature, ancestry or population affinity, and any unique identifying features. During the examination of human remains, it is important to be aware that the skeletal features considered when applying anthropological methods may be influenced and modified by a number of factors, and particular to this article, prescription drugs (including medical and non-medical use) and other commonly used drugs. In view of this, this paper aims to review the medical, clinical and pharmacological literature to enable an assessment of those drug groups that as side effects have the potential to have an adverse effect on the skeleton, and explore whether or not they can influence the estimation of age-at-death, sex and other indicators of the biological profile. Moreover, it may be that the observation of certain alterations or inconsistencies in the skeleton may relate to the use of drugs or medication, and this in turn may help narrow down the list of missing persons to which a set of human remains could belong. The information gathered from the clinical and medical literature has been extracted with a forensic anthropological perspective and provides an awareness on how several drugs, such as opioids, cocaine, corticosteroids, non-steroidal anti-inflammatory drugs, alcohol, tobacco and others have notable effects on bone. Through different mechanisms, drugs can alter bone mineral density, causing osteopenia, osteoporosis, increase the risk of fractures, osteonecrosis, and oral changes. Not much has been written on the influence of drugs on the skeleton from the forensic anthropological practitioner perspective; and this review, in spite of its limitations and the requirement of further research, aims to investigate the current knowledge of the possible effects of both prescription and recreational drugs on bones, contributing to providing a better awareness in forensic anthropological practice and assisting in the identification process of the deceased.

## 1. Introduction

Amongst the requests forensic anthropologists undertake, one major role is to assist in the identification of the deceased through primarily the analysis of human skeletal remains [1,2,3,4]. In this regard, during the post-mortem examination of the remains, the anthropologist may be asked to provide information on the biological profile of the individual; this can include the estimation of age-at-death, sex, stature, ancestry (or population affinity), and identifying any unique features [5]. Age-at-death estimation may involve the assessment of skeletal maturation, dental development, and morphological changes in areas such as the pubic symphysis, the rib end, and the auricular surface of the ilium [6,7]. Biological sex estimation may involve an analysis of the pelvic bones, the skull, possibly complemented by metric data [8]. Stature will be estimated by applying bone measurements to an equation [9]; whilst ancestry may be estimated using morphoscopic or metric analyses [10,11,12]. The skeleton will also be examined for any identifying features such as non-metric traits, evidence of surgery and pathological conditions, that may assist in narrowing down the list of missing persons whose remains are being analyzed [3,5,13]. 

However, it is important to remember that skeletal indicators considered for the reconstruction of the biological profile are influenced by a number of factors including age, sex, disease, genetics, lifestyle, diet, and pertinent here, possibly the use of prescription drugs (medical and non-medical) and other commonly used drugs, such as those drugs of abuse. Indeed, the medical literature describes how various drugs can affect the skeleton [14] and thus modify characteristic bone quality, appearance, shape and size of skeletal areas [15], which are used for the reconstruction of the biological profile. 

The United Nation Office for Drug and Crime estimates that about 275 million people worldwide made use of drugs at least once in 2019, a number that has been increasing by the millions in recent years [16]. Moreover, according to the World Health Organization (WHO), drug use led to approximately 450,000 deaths in 2015 [17]. These figures, added to the number of people that regularly take (prescribed) drugs for medical reasons, show the scale of the phenomenon and in turn the importance of considering the impact of drugs on the skeleton during forensic anthropological casework. 

This theme has not been thoroughly investigated in the context of skeletal analysis in forensic anthropology. To date, published literature in this area has so far explored only a minimal part of these effects. For instance, the investigation of particular bone manifestations of cocaine abuse trough CT scans [18]; discussing how homeostasis can change due to alcohol and drug use, affecting the ability to accurately assess estimation of age-at-death [19,20]; or experimental approaches with human analogues on opioids [21]. The presence of drugs in bones has been studied mainly in skeletal toxicology, where the substance is detected analytically [22,23,24,25,26,27], but very little has been done macroscopically with imaging or by direct examination of the bones. 

The main aim of this paper, therefore, is to present and discuss the potential skeletal effects of different medications and drugs based on a review of the literature. This has two advantages: (1) to consider these possible effects when assessing the biological profile through the estimation of age-at-death, sex, stature, etc. from the skeleton; (2) explore whether any changes to the skeleton may be specific to a particular drug or class of drugs, which may then in turn assist with identification, in particular if the medical history of the deceased is available. Although this review is not exhaustive, the final overall aim is to also provide an awareness for the forensic anthropological practitioner, and highlight the importance of further research on this topic.

## 2. Materials and Methods

To achieve the aims of this paper, medical, clinical, pharmacological and forensic anthropological literature was researched in several scientific databases; and scientific journals and medical books were accessed. The analysis of the literature was divided into two steps: first, the general relationship between drugs and bone health was investigated; second, specific research was carried out on the different drugs that may have bone involvement as side effects. 

The literature search was performed between November 2019 and October 2021 and built from a previous MSc thesis [28], using the keywords “bone/s”, “drug/s”, “medication/s” on several databases including PubMed (Medline), Scopus, Science Direct and Web of Science, as well as Google. Once specific drugs were identified, a more directed research was run using their names to further investigate their effects on bones and a number of drug databases were consulted including Vademecum (www.vademecum.es, accessed 28 October 2021), the Spanish Agency for Medicine (CIMA https://cima.aemps.es/cima/publico/home.html#quees, accessed 28 October 2021), Substance Abuse and Mental Health Administration (SAMHA https://www.samhsa.gov/, accessed 28 October 2021), Alcohol and Drug Foundation (ADF https://adf.org.au/drug-facts/#list, accessed 28 October 2021), National Cancer Institute (https://seer.cancer.gov/seertools/seerrx/, accessed 28 October 2021), UK Government website (https://www.gov.uk/guidance/find-product-information-abut-medicines, accessed 28 October 2021), and the US Food and Drug Administration (FDA https://www.accessdata.fda.gov/scripts/cder/daf/index.cfm, accessed 28 October 2021). Moreover, the Prescription Drugs and Over-the-Counter (OTC) Drugs identified, the official product label was reviewed to check whether the suspected adverse reaction was consistent with those described in the product label (The European Summary of Product Characteristics (SmPC) and the United States Prescribing information (USPI). No restriction regarding the date of publication was applied. Teeth and oral health were examined briefly as this is the remit of the forensic odontologist, rather than the forensic anthropologist. 

The results were summarized and organized in two tables by class and type of drug, showing their reported effect on bones and if any, the area of the skeleton most commonly involved. In addition, it was reported whether they could potentially affect sex and age estimation or any other biological profile parameter. 

## 3. Results

The information collected from the literature shows that commonly used drugs (with the potential for misuse or addiction such as prescription opioid, tobacco and alcohol), prescription drugs and even over-the-counter drugs, if taken long term and/or in high doses, have the potential to cause numerous health issues, including bone modifications at different levels [14]. 

The most commonly used drugs (with the potential for misuse and addition), defined as psychoactive drugs, can be categorised as stimulants, narcotics (opioids), depressants, hallucinogens and marijuana (cannabis) [29]. As will be seen in later sections of this paper, among stimulants, the principal drugs that can have a detrimental effect on the skeleton are cocaine, amphetamines, and nicotine (i.e., the main component of tobacco). Opioids include morphine and its derivatives, methadone and heroin. Alcohol and others (such as benzodiazepines and barbiturates) are depressant drugs with proved side effects on bones, while amongst hallucinogens, ecstasy can also be associated to bone disease. Opioids can be prescription medications, and along with some over-the-counter medications (i.e., nonsteroidal anti-inflammatory drugs and paracetamol), can lead to addiction and are commonly abused. However, side effects which affect the skeleton can also occur by taking controlled doses of other prescribed drugs that usually do not cause addiction but are extensively used in clinical medicine. These medications include corticosteroids, antiresorptive drugs, gonadotropin releasing hormones (GnRH) agonists, gastric acid suppressants or proton pump inhibitors, thyroid hormones and antiretroviral, antidepressant, antipsychotic, antiepileptic, antidiabetic, and antithrombotic drugs. These are included in more detail in the following section.

### 3.1. Effects of Drugs on Bone

This section includes the drug classes that, as a result of the research undertaken for this paper, can have an adverse effect on bone. This paper avoids brand names or trademarks and mainly provides classifications that are either generic or according to effect (therapeutic classification), chemical components or mechanisms (pharmacological classification). Brief definitions are provided, alongside a brief overview of their use and how they can affect the skeleton. For each drug, and whenever applicable, macroscopic bone lesions are described as well as their potential effect on the process of age-at-death and sex estimation in forensic anthropology practice. This review is not extensive, at least in its bibliography, but it provides an insight into how medication and drugs of abuse can modify the skeleton, which is an important consideration for forensic anthropologists. A small mention to dental disease and oral pathology, as well as cartilage, is included at the end. Limitations and interpretations are discussed later.

#### 3.1.1. Cocaine 

Cocaine is an alkaloid derived from the leaves of the *Erythroxylum coca* plant. It is currently used as an intraoperative local anaesthetic and vasoconstrictor, but it also represents one of the most common drugs of abuse [30]. Recreational cocaine is often contaminated by various additive compounds, such as levamisole, which can be directly responsible for the effects of the drug and/or its local and systemic complications, or act as a contributing factor [31,32]. Cocaine can be administered through intravenous injection, nasal insufflation (the most common), inhalation (smoking), direct application on mucous membranes or chewed and rubbed on the gum. The way cocaine is administered will influence the effect of the drug on bones [30]. In fact, the intranasal use (insufflation) is responsible for one of the most important effects of cocaine on bones, the cocaine-induced midline destructive lesion (CIMDL), characterized by the destruction of the nasal septum, lateral nasal walls and/or hard palate [33,34,35]. Rubin [18] defined this condition as any significant bone damage of the midfacial region clearly caused by the use of cocaine and identifiable in human skeletal remains. Its pathogenesis is mainly related to the vasoconstrictive effect of cocaine, leading to ischemic necrosis, combined with the chemical irritation of adulterants, direct trauma from the use of paraphernalia and possible superinfection [34]. Thus, after repetitive and frequent snorting, the blood vessels of the nasal mucosa become atrophic and irritated, resulting in localised ischemia and ultimately in necrosis, erosion and destruction of the osteocartilaginous tissue. Septal perforation tends to be observed first, and the lesion then progresses and involves the nasal lateral walls with saddle-nose or alar deformities, the hard palate with oro-nasal fistulas, and even the maxillary sinuses and orbital walls due to chronic inflammation and infection of the sinuses [36,37,38]. Rubin [18] considers how forensic anthropologists should consider someone as a cocaine abuser where there is lack of new bone formation to repair the lytic lesions. These destructive lesions are primarily located in the vomer, in the palate (palatine bones) and inferior nasal conchae; with other bones affected being the ethmoid, maxillary sinuses, sphenoid and orbit [18]. One clinical case showed also an extension of CIMDL into the neck area, especially with some destruction and instability of the atlanto-axial joint [39].

#### 3.1.2. Opioids

These are naturally found in the opium poppy and can be prescription medications often referred to as painkillers, although are often used non-medically or recreationally. Their use is widespread, and data has shown that it has been taken illegally since adolescence [40]. Three most commonly used opioids are covered here: morphine, methadone, and heroin.

The use of morphine to manage chronic pain is widespread. However, as it would appear that it inhibits osteoblastic activity and certain hormones such as gonadotropin-releasing hormone (GnRH) [41,42], it has been shown that opioids can induce osteoporosis and thus increase osteoporosis risk fracture [43]. This reduction in bone density and thus leading to osteoporosis has been demonstrated in some human and non-human experimental studies [44], although other factors, leading to this lower bone mass density, need to be considered [45]. The risk of fracture in morphine users also increases, especially in common osteoporotic fractures such as those found at the hip, spine, and forearm; a risk increased by loss of postural balance and falls due to side effects of the drug [46]. This, in turn, although not with all opioids, leads negatively to bone healing, and bone non-union may result [47]. Moreover, as it affects cell proliferation and apoptosis [48], experimental studies on rats have shown that morphine in mothers have effects on the primary and secondary ossification and longitudinal growth of their offspring [48,49]. 

With regard to methadone, Kim and colleagues [50] investigated the low bone mineral density (BMD) in patients taking part in a methadone maintenance program in Boston. Using dual energy x-ray absorptiometry (DXA) combined with surveys and medical records, the study found that BMD of 83% of the study sample were below normal, with 35% of those within the osteoporosis range, and 48% of those in the osteopenia range. This in turn, resulted in a higher fracture risk for those who were taken methadone [51]. Similar studies have been undertaken on male and female subjects yielding different results, with more significant bone loss in the former than in the latter [52,53]. This association may be related to the effect opioids have on bone metabolism, in particular inhibiting osteoblastic (bone formation) activity [54]. 

Heroin is made by adding two acetyl groups to the molecule morphine. As heroin can alter several body functions, chronic abusers present with altered bone metabolism and reduced trabecular bone mass, which according to Pedrazzoni et al. [55] is attributable partly to hypogonadism. Wilczek, H., and Stĕpán, J. [56] investigated the effects of prolonged use of heroin and noted, focusing on the femoral neck and forearm, that it is associated with accelerated bone turnover, resulting in osteopenia. However, after one year of treatment with methadone, bone turnover rate was restored. In addition, a Spanish study noted the presence of septic arthritis in heroin users, affecting especially the sacroiliac, costoclavicular, hip and shoulder joints [57]. In fact, intravenous drug injection in heroin addicts has been associated with osteomyelitis. In a study by Allison et al. [58], out of 215 patients injecting drugs, 59% had osteomyelitis and 25% septic arthritis. In fact, septic arthritis at the pubic symphysis has been found to have intravenous drug injection as a risk factor [59]. Similar associations with osteomyelitis have been found in other studies in the last decades where joint disease and infectious skeletal lesions have been present, usually in the limbs and sites where the injections have taken place [60]. A number of cases since the 1980s have also reported cervical osteomyelitis in intravenous drug use [61,62]. 

There are also other drugs in this group, such as Desomorphine, a synthesized opioid from codeine which has been associated with skeletal infections at the site of skin ulcers due to injection, followed by necrosis and gangrene in some cases, and amputation [63,64]. Due to the toxic substances in the manufacturing process of this highly addictive drug, as well as the injectable equipment and hygiene, the risk of infection is much larger and more severe than that of any other drug with the same administration [64]. Some of these drugs have also shown to cause necrosis of the mandible and maxilla [65,66]. 

#### 3.1.3. Amphetamines 

As stimulants, they speed up the transmission between the brain and different parts of the body. There are different types of amphetamines, some being prescribed to treat disorders such as attention deficit hyperactivity disorder (ADHD) and other conditions (https://www.dea.gov/sites/default/files/2020-06/Amphetamines-2020_0.pdf (accessed 29 October 2021)). The most potent form is methamphetamine (METH). The main route of administration is orally, but can also be injected intravenously, or taken by insufflation, inhalation and suppository. Amphetamines decrease bone mass and strength due to the drug effect on the central nervous system, closely linked to bone metabolism and affecting bone turnover [67]. A strong correlation has been found in the literature between methamphetamine users and lower bone density and osteoporosis [51]. For example, Katsuragawa [68] found a decrease in bone mass and integrity in the calcaneus of drug users. In addition, Mosti and colleagues [69] examined loss of bone density by assessing whether it was localized (specifically, to the hip or lumbar spine), or generalized. The study found a general loss of bone density through DXA scans and also a reduction in lower limb muscle strength [69]. A number of reported cases, have also found that apart from loss of bone density, osteonecrosis or osteomyelitis can be found in the jaw [70], as well as maxillary sinusitis [71]. Any effects on dental and oral health are reported in a separate section below.

#### 3.1.4. Cannabinoids

Cannabinoids are the chemical components found in the Cannabis plant (Marijuana). The main psychoactive chemical is tetrahydrocannabinol (THC). The drugs can be smoked, inhaled or eaten. Cannabis (marijuana) or hemp are legally accepted in some regions and countries as they also demonstrated health benefits [72]. Indeed, the chemical components activate the endocannabinoid receptors of the body and brain resulting in a feeling of happiness, but they can also affect bone homeostasis [72,73] (http://www.thedrugswheel.com/; https://adf.org.au/drug-facts/cannabinoids/ (accessed 29 October 2021)). Studies have shown a significant decrease in bone mass density and bone quality among smokers of marijuana with respect to non-smokers [74]. Paradoxically, depending on the age of the individual, cannabis can also help with bone loss and has been used to manage osteoporosis [75]. However, no correlation was found between cannabis consumers and bone density in a study on the femur and lumbar spine in a U.S. study [76]. The positive and negative effects are still unclear at present [72,77]. The effects of Marijuana on teeth is covered in a separate section below.

#### 3.1.5. Alcohol

Alcohol is a depressant like diazepam or benzodiazepines, thus slowing down the message between the brain and the body, and hence its vital functions. Depending on the amount taken and body composition, however, it can also act as a stimulant. A number of publications have examined the association between alcohol and bone disease in adolescents and adults [78,79,80]. The effects of light consumption, long-term and binge-drinking have been investigated in clinical studies [79]. It has been demonstrated that alcohol can affect bone proliferation and lead to low bone density (leading to osteopenia and possibly osteoporosis) and strength due to a remodeling imbalance [81,82,83]. However, this is dependent on the pattern of consumption and intake [84,85]. One study revealed that 12% of fractures in middle-aged men, could be avoided if alcohol, as well as smoking, were eliminated [86]. Alcohol can also inhibit osteoblast proliferation and thus be detrimental to fracture healing [87]. One paper in forensic anthropology suggested that an individual’s age-at-death may have been overestimated from the skeletal remains of a person who suffered from alcoholism. The case presented cortical thinning, ‘light’ bones, as well as various skeletal fractures in different stages of healing; although these characteristics may more likely be secondary to alcoholism than due to the age of the individual [20]. Furthermore, osteonecrosis associated with alcoholism has been identified and widely reported in the clinical literature, especially avascular necrosis of the femoral head [88,89]. Much information is also available relating to alcohol and pregnancy, which is not covered in detail here, but it is worth mentioning a number of skeletal anomalies affecting cranial suture such as craniosynostosis in the fetus due to alcohol consumption during pregnancy [90]. 

#### 3.1.6. Tobacco

There has been much research on the impact of smoking (nicotine and tobacco) on health, some of which has focused on bone health [91,92]. Amongst the skeletal complications caused by smoking are lower BMD [93,94] although this is still debatable [95,96,97], higher fracture risk [97], and delayed bone fracture healing and further complications [98,99,100]. A study on young adult (18-19 years) men, smokers vs. non-smokers, showed a reduction in BMD and also reduced cortical thickness in radius and tibia [101]. This in turn leads in smokers to an increase in fractures, especially osteoporotic fracture sites such as the spine, hip, wrist or major long bone shafts, but not to the skull [86]. Scolaro et al. [102] further demonstrates complications with fracture healing and nonunion in some instances. This delayed healing may be related to poor bone mineralization and smoking impairing Type I collagen fibrils [103] as well as other factors [104]. Complications of smoking on oral health are explored later, as well as in cartilage [105,106]. Pathological conditions may also be considered as a result or in association with tobacco, for instance an increase in degenerative joint disease in the vertebrae [107] or children in a smoking intrauterine and post-uterine environment where their skeletal growth and development may be affected [108]. 

#### 3.1.7. Oral Glucocorticoids

Glucocorticoid-induced osteoporosis is the most common iatrogenic cause of secondary osteoporosis. The direct effect that this class of drugs has on the skeletal structure is drug-induced osteoporosis if used long-term [109]. These drugs also affect the endocrine system, which controls a number of different hormone mechanisms, causing disorders such as hypogonadism, which again can affect bone turnover and decrease BMD [110]. Glucocorticoids are a class of corticosteroids, which regulate the metabolism of glucose in the body and are widely used in the medical sector for conditions that are caused by inflammation, such as asthma, allergies, auto-immune diseases and sepsis [111]. Prolonged or incorrect use of these can result in osteoporosis, osteonecrosis, high fracture risk and slower fracture repair [109,112]. Slower fracture repair especially callus formation and healing has also been observed in mice [113]. In children, glucocorticoids will result in short stature, delayed growth and maturation, unless reversed with growth hormone therapy [114,115]. This delayed growth can occur within three months of treatment with glucocorticoids and skeletal deformity may result from long-term treatment in children [116]. It may delay carpal bone age as observed in a Chinese study [117], a consideration relevant if estimation age in the living [118]. 

#### 3.1.8. Non-Steroid Anti-Inflammatory Drugs (NSAIDs)

Non-steroidal anti-inflammatory drugs (NSAIDs) are some of the most prescribed medications worldwide, with analgesic, anti-inflammatory, antipyretic and platelet antiaggregant functions [119,120]. This heterogeneous group of drugs acts by blocking cyclooxygenase enzymes (cox-1 and cox-2), which in turn inhibits prostaglandins synthesis, which has an important role in bone turnover by influencing both osteoblast and osteoclast activity [121,122,123]. Several studies have explored the effects of NSAIDs on fracture healing, as these drugs are commonly used for fracture and postoperative pain control following orthopaedic surgery [124,125]. However, some of these studies report how NSAIDs may delay bone healing [119,126,127,128,129,130]. An increased incidence of nonunion fractures, malunion and infections are observed, with examples of case reports of this in the femoral shaft and the spine [120,125,131,132,133,134]. However, some of this data has been extrapolated from animal studies, while human trials have not always reported strong evidence of this association [87,124,135,136]. NSAIDs also seem to impair entheses (tendon-to-bone) healing [123] and accelerate cartilage degeneration in osteoarthritis [137,138]. Regarding skeletal trauma, not all NSAIDs have been found associated with an increased risk of fractures [139]. For instance, diclofenac and naproxen have been associated with an increase fracture risk in hip, spine, and forearm; while others showed either a higher BMD, with a potentially lower fracture risk [136,140]; or did not show any association (e.g., aspirin) [119,141,142,143]. This positive effect on BMD (total and hip) was observed with increasing doses, whereas it decreases at low doses, potentially increasing the fracture risk [134,139,144]. In paediatrics, no effects on bone have been reported on low dose and short duration therapy [129,145]. By contrast, if chronically prescribed during pregnancy, and depending on the gestation period, NSAIDs may have adverse skeletal effects on the fetus and newborn, including presence of cleft palate, decreased skeletal development, decreased vertebral and fracture callus mineralization, decreased fetal length, fused ribs, incomplete ossification of the cervical arch, deformation of lumbar arch, and absent sacral arch [146]. 

#### 3.1.9. Paracetamol

Paracetamol (acetaminophen) is a drug with analgesic, antipyretic and mild anti-inflammatory properties, and is one of the most used medications worldwide [147]. Its mechanism of action involves the cyclooxygenase (COX) and cannabinoids pathways, decreasing prostaglandins production and in turn affecting bone turnover [140]. However, despite its very wide usage, very few studies have explored the potential link between this drug and bone health [148]. Changes in BMD and bone fragility with an increased risk of fractures have been the most studied [143]. Several authors have reported no difference in BMD between paracetamol users and non-users [147,149]. No significant differences were found according to dose and pattern of users (intermittent vs. continuous) [143]. By contrast, other studies have shown a decrease in BMD over time, although smaller than other analgesics such as NSAIDs and opioids [150]. Similar results are found when investigating the risk of fractures [139,140,143]. The risk of fracture has been reported for the spine, hip, and forearm, and it is not dose-dependent [139]. Moreover, the effects of this drug on proliferation and differentiation of osteoblasts, if used in the early phases of healing, may impair bone regeneration and implant osseointegration [148]. In contrast, other studies have not supported this association, for example Vestergaard et al. [143] detected slightly higher levels of alkaline phosphatase, a marker of bone turnover. Since conflicting results have been found so far on the effects of paracetamol on bone, and little is known about them [143], further studies are therefore needed to better investigate and understand the impact of this drug on bone health [140].

#### 3.1.10. Gonadotropin Releasing Hormone Agonists (GnRHa)

Gonadotropin releasing hormone agonists (GnRHa) are commonly used for treatment of several conditions, including breast cancer, prostate cancer, endometriosis, gender dysphoria and central precocious puberty (CPP) [151,152]. They act on the pituitary-hypothalamic-gonadal axis inducing secondary hypogonadism and reducing the production of sex steroid hormones in both sexes, oestrogens in women and androgens in men [152,153]. These hormones influence osteoblasts and osteoclasts activity, with important functions in bone turnover including bone growth and maturation [154,155]. Due to sex hormones deprivation, bone turnover is accelerated with suppressed bone formation and increased bone resorption. Therefore, GnRHa may have a detrimental effect on bone health causing reduction of BMD and increasing the risk of osteoporosis and fractures, as reported by several studies [153]. GnRHa are extensively used as adjuvant endocrine therapy in breast and prostate cancer [152], leading to a cancer treatment-induced bone loss [154]. This accelerated bone loss involves trabecular bone (spine) and is greater in women than in men ([152], resulting in a BMD reduction estimated between 5% and 10% in spine and hip after one year, and continuing to decrease in long-term therapy ([153]. GnRHa therapy also increases the risk of osteoporosis and fractures, with a longer therapy duration and a higher number of doses predicting a greater risk [156,157]. In women, lumbar spine and femoral neck fractures are the most commonly affected. In men, the radius, vertebra and hip/femur are the most frequently fractured bones [152]. GnRHa have been used to reduce pelvic pain, but this in turn has shown to lead to a reduction in BMD in the lumbar spine, hip/proximal femur and radius after 6 months of treatment, sometimes followed by a partial or complete recovery after a withdrawal of 6 months-1 year [155,158,159,160,161]. Differences have also been observed between different GnRHa, with leuprolide acetate having a greater detrimental effect on BMD than buserelin for example [155]. Whilst short-term therapy would unlikely cause bone loss, little data is available on the long-term consequences with regard to low BMD and fracture risk [155]. These drugs are also used in gender dysphoria and CPP in children and adolescents. The effects on bone are of concern due to the hormonal suppression occurring in puberty [162], potentially delaying or attenuating peak bone mass (PBM) although this is still not fully understood [163]. A decrease in BMD was observed in lumbar spine and femoral neck in transgender individuals [163,164] as well as in CPP, but with the latter showing reversible effects after withdrawal [151,165]. Nonetheless, attaining a normal PBM does not seem to be impaired [162]. 

### 3.2. Proton Pump Inhibitors

Proton pump inhibitors (PPI) are considered relatively safe and are widely used as acid-suppressor medicine to treat acid-related diseases (e.g., gastroesophageal reflux, peptic ulcers, heart-burn, dyspepsia, chronic cough, prevention of gastric injuries from NSAIDs and surgery) [166].). They are a class of drug that act on the cells that line the gastrointestinal tract and reduce acid production, allowing the lining to heal, or to prevent an ulcer from occurring [167]. There is a large body of evidence that demonstrates an association between PPI therapy and risk of fractures, in particular a moderate increased risk of any fractures in particular to the hip and spine, with a stronger association of hip fractures with increased duration of PPI treatment, as well as an association between PPI therapy and osteoporosis [166,168]. The association between PPI use and BMD is debatable, with some studies showing BMD loss [169] and others concluding an absence of correlation [166,170]. Two main factors may explain the association between PPI therapy and increased fracture risk as well as osteoporosis. Firstly, decreased calcium absorption has been noted in patients taking PPI, which would cause an increased rate of bone resorption; however, there are various factors, which may influence calcium absorption (e.g., dietary calcium intake and time of medication) [166]. Secondly, a selection bias and the absence of adjustment for cofounders (which include a large number of comorbidities and medication): older and sicker patients tend to be treated with PPI, and frailty and old age are risk factors for fractures [166,168]. 

### 3.3. Antiretroviral Therapy 

Antiretroviral therapy (ART) are drugs that are taken to treat and prevent mortality and morbidity by retrovirus infections, such as human immunodeficiency virus (HIV). These drugs help control the virus by lowering the viral load, preventing transmission, and increasing life-expectancy rather than actually curing the disease [171]. Whilst there may be about a dozen drugs to treat HIV, it is a combination of these that are prescribed for therapy. HIV is already known to affect the skeletal system through low BMD, osteoporosis, osteonecrosis and more rarely, osteomalacia, as well as fractures and HIV-induced infections and inflammations [172,173,174]. Osteonecrosis is commonly present in the proximal femora and may be bilateral [175,176]. (Regarding ART, several studies have demonstrated an association between long-standing ART and lower BMD in HIV individuals [173,174,177,178,179], although other research reported no determining effect of ART on BMD [180]. Overall, low BMD in HIV patients results from a multifactorial interaction between HIV infection, conventional risk factors for osteoporosis, ART-related complications and HIV/AIDS-related conditions (e.g., muscle wasting, kidney disease, vitamin D deficiency and hypogonadism) [177,181,182,183]. In addition to low BMD, both long-standing HIV and ART have been reported to be associated to osteopenia, osteoporosis, osteonecrosis, osteomalacia and a higher rate of fractures [173,179]. Indeed, ART has a direct effect on the bone metabolism by exacerbating bone loss (with a reported 2–6% loss in BMD) at the femora, lumbar spine, and hips; which are sites susceptible to fractures [173,179,183]. Lastly, neuropathy may be another potential complication of ART [184,185], which may indirectly impact the skeletal system by leading to conditions such as neuropathic arthropathy (Charcot joint) [186,187].

### 3.4. Anti-Depressant Drugs

Patients that suffer with depression often have low levels of serotonin, which is a neurotransmitter found mainly in the gastrointestinal tract, platelets and the central nervous system (CNS) and is a contributor to feelings of wellbeing and happiness (InformedHealth.org (internet). Cologne, Germany: Institute for Quality and Efficiency in Health Care (IQWiG); 2006. Depression: how effective are antidepressants? (Updated 18 June 2020) (accessed October 2021)). In some countries they are the most used therapeutic medications [188]. It also regulates the skeletal response to parathyroid hormone due to its receptors that are found on osteoblasts and osteocytes. Two commonly prescribed classes of drugs are selective serotonin re-uptake inhibitors (SSRIs) and tricyclic anti-depressants (TCAs) [189]. This paper focuses mainly on SSIRs, which seem to be associated to bone metabolism [190,191]. Bone loss density, rapid bone loss in certain age groups and an increase in osteoporosis in men has been shown in those taking anti-depressant drugs [192,193,194] and thus a risk of osteoporotic fracture [195,196]. Furthermore, in an experimental animal study, sertraline was shown to impair and disrupt bone healing with significant decrease in trabecular thickness ([197,198].

The link between fracture risk and SSIRs has been widely noted, however, depression itself has been shown to correlate with a decreased bone mineral density and increase fracture risk [199]. Although, taking into consideration the psychological condition of the individual receiving treatment, there is a likely chance that there will be other lifestyle risk factors, which may influence bone mineral density and increased fracture risk, such as smoking, increased alcohol consumption and physical inactivity [189]. Thus further work is required to show any link with depression, drugs and bone health [193,200]. 

### 3.5. Anti-Epileptic Drugs

Chung & Ahn [201] discuss the effects of anti-epileptic drugs (AED) and their effect on bone in children being treated for epilepsy. The authors examined bone density scans on a number of skeletal areas including the upper and lower limbs, the ribs, pelvis, and spine in a sample of 78 epileptic and 78 control patients, and concluded that the former group, which was treated with AED, had lower bone density. Lower bone density in those taking AED seems to correlate in other studies for different anatomical regions [202,203,204]. Other studies in adults have shown no known significant differences between short-term and long-term use of these drugs in the overall skeleton, but significant differences when specific bones are taken into account, such as the tibia and innominates [205]. It has been suggested that the reason for this lower BMD is that anti-epileptic drugs directly inhibit osteoblast function as well as inhibiting intestinal calcium absorption [109]. This reduction in bone mass density also increases fracture risk. In adults, the association with osteopenia and osteoporosis has been demonstrated [206,207], with increased fracture rates associated to the drugs as well as the result of seizures. Although the results are conflicting [208], generally speaking these drugs will lead to low bone density as well as an increased risk of fractures [209,210]. Reduced levels of Vitamin D have also been observed with AED intake [211,212] and also retarded growth and stunting [213].

### 3.6. Antidiabetic Drugs

These medications, including insulin, exist to control and maintain glucose or sugar levels in the blood and thus more commonly used to manage diabetes, adversely affect bone metabolism [214], especially by impairing osteoblast function and activating osteoclastogenesis [215]. This may ultimately lead to a decrease in low bone mineral density, decrease bone strength related to low bone turnover, alteration of the microstructure, and a risk of osteoporotic fractures such as at the hip [216,217]. This is of course also drug type dependent [218,219]. For example, thiazolidinedione in particular is associated with secondary osteoporosis and an increased fracture risk [219,220,221]. Overall, antidiabetic drugs are linked to an increase risk of osteoporosis, fractures and possibly osteoarthritis too [218,222,223], although this latter is not yet clear [224].

### 3.7. Antiresorptive Drugs

These drugs include a class termed bisphosphonates. These inhibit osteoclastic activity and although bisphosphonates are likely to control osteolysis in tumors and disease progression [225,226], they also do have other effects, for instance osteonecrosis of the jaws and more frequently in the mandible [227,228]. Osteonecrosis of the jaw (ONJ) is a well-known complication of antiresorptive or antiangiogenic therapy for the management of osteoporosis and other cancer-related conditions [229]. Available data indicate that 5% of patients exposed to antiresorptive agents may develop ONJ, depending on the duration of therapy. Oral surgical procedures, tooth extractions and infection of the mandible and/or maxilla are considered the main risk factors for developing ONJ when receiving antiresorptive therapy [228]. A study by Gupta and Gupta [227] indicates that osteonecrosis tends to develop in the jaw because it has a higher remodeling rate than other bones, making it more prone to the effect of bisphosphonates. The three most common sites for ONJ are (1) nonhealing dentoalveolar sites or dental extraction sites; (2) traumatized tori (palatal and/or mandibular); and (3) exposure of portions of the mylohyoid bridge [227,230,231]. Osteomyelitis and abscesses may also be present and in living individuals exposed bone too [231,232].

Bisphosphonates with denosumab are the most commonly used antiresorptive drugs and although they cause osteonecrosis of the jaw [233] when used to treat malignant disease, they are used to treat osteoporosis and the risk of fracture associated from it [234,235].

### 3.8. Antithrombotic Drugs

Antithrombotic drugs can be antiplatelets (e.g., aspirin) or anticoagulants (e.g., heparin, warfarin) and prevent blood clots from forming. A number of groups would appear through a literature review to affect bone health, primarily linked to osteopenia [236]. Some anticoagulants such as heparin may result in lower bone mass density, influencing bone metabolism and resulting in an increased risk of osteoporotic fractures [237,238]. Impaired fracture healing may also take place [239]. One study on warfarin demonstrated an association with a decrease in BMD in the calcaneus of patients compared to non-patients through examination with a quantitative ultrasound [240] 

### 3.9. Other Drugs

This paper has not covered all drug groups, all the different classes of drugs, nor the combination of taking several classes together and how this may affect the skeleton. Nevertheless, it is worth mentioning here a number of other drugs that may have a significant effect on the skeleton too. For example, Depot Medroxyprogesterone Acetate (DMPA) or Depo-Provera is a contraceptive drug that both adult and adolescent females may take. It works by inhibiting luteinizing hormone (produced and released by the pituitary gland) and follicle stimulating hormone (also released from the pituitary gland and important for the reproductive system in men and women), which in turn decreases oestrogen production [118], thus resulting in decreased bone mineral density and increase risk of osteoporosis during its use. Most bone loss occurs in the first two years of use and mainly seen in the vertebral column and hips – and so this is where most fractures are seen. A study conducted using a group of physically active female army recruits indicated that there was a marked increase in stress fractures in the calcaneus of the individuals taking DMPA [109]. 

Amongst the hormone therapy drugs, aromatase inhibitors (AI) which has been used to treat a number of diseases such as breast cancer, does result in bone loss and a risk of fractures [241,242]. Another hormone treatment is thyroid hormone therapy (THT), which is used to compensate for an underactive thyroid. Patients with hypothyroidism undertaking THT may or may not see skeletal changes. The literature provides conflicting reports on BMD, with some studies showing BMD loss while others found no changes [243,244,245]. 

Antineoplastic drugs are chemotherapy drugs and highly toxic but used to treat cancer. Since there are almost 2000 medications under this class of drug (https://seer.cancer.gov/seertools/seerrx/, accessed 31 October 2021), it is impossible to cover here, especially when in different forms. It is worth indicating that some side effects will include lower BMD, bone marrow suppression, haematological complications including anaemia, periapical lesions possibly leading to osteomyelitis, etc. [246,247,248,249,250].

It is also worth mentioning antipsychotic medications or agents, used to manage and/or treat patients with psychosis (e.g., in patients with schizophrenia, bipolar disorders, etc). Antipsychotic drugs have a physiological effect on bone, as they increase the concentration of prolactin, which lowers oestrogen and testosterone levels potentially leading to bone loss [251]. One study indicated that the risk of a hip fracture was increased 5-fold in older women and 6-fold in older men taking antipsychotic drugs [109]. In one study, it was also noted that in young men and pre-menopausal women these drugs lower bone mineral density as much as 20% in the spine [252]. 

### 3.10. Oral Pathology 

Although already introduced above, it is worth providing an overview of the dental and oral (bone) complications that can arise in patients taking some of these drugs. In forensic cases, this is the remit of the forensic odontologist, but nevertheless it is important for forensic anthropologists to be aware of these changes, in addition to other factors that may affect oral pathology such as lifestyle or oral hygiene practices. 

Tomita et al. [67] indicate that in a very short space of time, rampant caries is often found in methamphetamine users (“meth mouth”). In addition to caries, periodontal disease and tooth loss [253], partly due to the reduction of saliva in the mouth and other factors [254]. Cocaine can also damage teeth and the surrounding soft tissue, as one common method for consumption is by rubbing the powder against the gums or gingivae. Cocaine reduces salivary pH leading to dental erosion, and there is a higher risk of periodontal disease and tooth loss [255,256,257,258,259]. Smoking or eating cannabis has also similar effects [260]. 

Tooth discoloration can be caused by medication such as antihistamines and antibiotics amongst others [261,262,263]. Furthermore, enamel hypoplasia as well as microdontia and hypodontia can be found in children treated with antineoplastic drugs [250]. Smoking tobacco can also cause tooth discoloration [264] although there are many other factors influencing color staining in teeth [265]. 

With regard to the alveolar bone and further involvement of some of the drugs included above such as anti-resorptive drugs and antineoplastic drugs can result in osteonecrosis of the jaw [63,64].

### 3.11. Other Skeletal Involvement

As the skeleton is also composed of cartilage and cartilage degeneration will affect some of the indicators forensic anthropologists use in reconstructing the biological profile, it is necessary to point out how some drugs and medications can affect cartilage. For example, smoking tobacco has been found to be associated with cartilage loss and defects in the cartilage of offspring [106]. This association resulting in osteoarthritis has also been proven in other studies. Amin and colleagues [105] found that men with knee osteoarthritis who smoke sustain greater cartilage loss and have more severe knee pain than men who do not smoke. One area of interest may be the calcification of cartilage, whether costal (sternal end of the rib) or any other (e.g., thyroid cartilage). Premature calcification of cartilage has been attributed to a number of aetiologies [266]. However, chondrocalcinosis as well as chondritis has also been attributed to certain drugs such as corticosteroids, bisphosphonates and others [267,268,269,270]. 

As forensic anthropologists during recovery of human remains, we may encounter urinary or renal stones or calculi. These can also be drug induced [271,272]. 

### 3.12. Summary of Results and Further Observations

Taking all the above information gathered from an exhaustive literature search in the medical, clinical, pharmacological and other disciplines; it can be understood that medication and the abuse of drugs can have an effect on the skeleton. These include loss of bone density often leading to osteoporosis and risk of fractures, necrosis, joint disease, delayed maturation, delayed fracture healing, cartilage calcification, and oral pathologies (Table 1). Whist some of these drugs may affect the skeleton generally, some studies have focused on particular regions or elements and some medications definitely only involve certain areas (Table 1), such as the vertebrae, long bones, mandible or maxilla. These may influence the estimation of the biological profile related to age-at-death estimation, sex estimation and other parameters or features used to identify the deceased. Table 1 should assist forensic anthropologists in their awareness of how certain medical histories and the associated use of certain drugs may affect the skeleton. This may be an important consideration when reconstructing the biological profile. In addition, some of these skeletal alterations may reflect a person that was using certain medication(s) and thus it may also be able to help with narrowing down the list of missing people.

Table 2 summarizes those drugs that particularly lead to loss of bone mineral density, potentially osteoporosis and risk of fracture. An additional column for bone destruction as also been included.

In addition, it is worth stating that apart from knowing the effects of drugs on bone there is potential to investigate these post-mortem through toxicological analysis of the bone. As drugs can be incorporated into bone through superficial arteries, born in the periosteal network, which later diffuse into the peripheral layer of the compact bone; or they can circulate through deep arteries and nutrient foramina toward the spongy bone tissue to terminate in the bone trabeculae and bone marrow; within the bone matrix, drugs can remain for instance in hydroxyapatite and be incorporated into the inorganic matrix through bone remodeling. Through these mechanisms, drugs can be preserved and detected in bone tissue even after a long post-mortem interval [18,27]. As evidenced in Table 3, toxicological analyses have been performed on various bone samples such as the cranium, rib, femur, vertebra, clavicle, and iliac crest [27,273,274]. As a result, the literature (Table 3) reports the detection of MDA (amphetamine), ketamine (anesthetic), pregabalin and carbamazepine (anticonvulsant drugs), diphenhydramine (antihistamine drug), atenolol and bisoprolol (antihypertensive drugs), caffeine, cocaine and its metabolite (stimulants), THCCOOH (metabolite of THC, a cannabinoid), laudanosine (metabolite of atracurium, a curare), as well as many antidepressants, antipsychotic drugs, benzodiazepines and opioids. 

Although further research is required, results have shown that these drugs can be detected years after death. 

## 4. Discussion

The aim of this paper is to increase awareness for forensic anthropologists on the effects that medication and drugs can have on the skeleton. This awareness will help with any considerations in forensic practice but it also opens new avenues for research. Prior to discussing the specific implications for biological profile and personal identification, a number of limitations need to be highlighted first.

### 4.1. Limitations

One of the limitations to highlight is that many if not most of these medications or drugs have similar effects on bones, and rarely are any of these changes pathognomonic to a specific drug, let alone other factors that can influence the alteration to the skeleton. For example, many of the drug classes described above will result in lower bone mineral density, and increase risk of osteoporosis and osteoporotic fractures. In turn, some drugs induce osteoporosis, for example, but osteoporosis can also occur as a natural disease. 

A second and important limitation is that this study has taken each class of drug separately. Whilst a person may take one specific medication, this paper has not considered a combination of different drug classes and its effect on the skeleton. For example, the consumption of opioids in addition to prolonged alcohol ingestion. Furthermore, the effects after drug intake must be examined in detail to assess how long before any effects are reversed. This is beyond the scope of this paper. 

Another difficulty in interpreting bone changes possibly related to drugs is that these may be influenced by a number of variables, including dosage, method of administration, and duration of treatment. All of these will have a different effect on the skeleton. One such example is cocaine, which if snorted, can cause destruction of the palatine and nasal bones [30]. The biological age of the individual as well as sex may also influence the effects on the skeleton. 

Similar to palaeopathology or pathological alterations to the skeleton, diagnosis will be reliant on bone preservation, bone condition, skeletal completeness, distribution of the lesions over the skeleton, if unilateral or bilateral, etc. In addition, if bone mineral density is to be observed it is likely that specific imaging techniques are necessary, rather than a direct visual assessments of the bones. 

As with many of the other drugs, a full understanding of each drug and its relation to the skeleton is not always clear, and is often dependent on age, sex and lifestyle factors. Moreover, to be more relevant to the forensic anthropologist, a more specific description of the skeletal lesions may be required, for example detailed information on osteonecrosis of the jaw including shape and dimensions of lesion, etc. 

### 4.2. Implications for Forensic Anthropology: Effects on Age-at-Death, Sex Estimation and Other Parameters 

As there has not been a published study in forensic anthropology regarding specifically how these drugs affect age and sex estimation methods, no definitive answer can be given. However, having observed some of the effects on the skeleton with some drugs, it can be hypothesized that some of these are likely to affect the indicators for age-at-death and sex estimation. Importantly, there may be issues around age estimation in the living, especially around skeletal growth. For age-at-death estimation, costal cartilage or pubic symphysis morphology may have been affected. For instance, if the individual presents with osteoporotic bones but is otherwise young, drugs such as corticosteroids, glucocorticoids, aromatase inhibitors and Depo-Provera could have resulted in this decreased bone density. Sex hormones may alter some sexual diagnostic features with more gracile bones and smaller muscle attachments, thus analysis of sex could be estimated incorrectly.

#### 4.2.1. Implications for Personal Identification

Alterations of bone mineral density, such as osteopenia and osteoporosis, and their consequent increased risk of fractures are the most common effects of drugs on bones as reported by the literature. However, due to their non-specificity, it is not possible to directly link these bone changes to the use of particular therapy drugs or drugs of abuse, as they could also be the outcome of normal ageing, other pathological conditions (such as endocrine disorders, eating disorders, immobilization, marrow-related disorders, disorders of the gastrointestinal or biliary tract, renal disease, and cancer) [281] and traumatic events; all potentially unrelated to the consumption of drugs. Table 4 proposes some possible influences of drugs in the reconstruction of the biological profile. 

In addition, results from forensic toxicology as seen in Table 3, in conjunction with skeletal changes that may be drug related, could help identification by adding to the biological profile. 

## 5. Conclusions

Given the number of people taking drugs (including drugs of abuse and prescription medication) today, the aim of this paper was to present the main drugs that according to the medical and anthropological literature consulted for this paper have the potential to affect the skeleton directly. Through an extensive literature review, the information was evaluated and extrapolated from a forensic anthropology perspective, considering the impact for the reconstruction of a biological profile when studying unidentified human remains; or at least increase an awareness of possible alterations of the skeleton due to drugs and medication. The list of drug categories included here is more generic and does not address particular names of drugs or brands, or venture in any detailed characteristics of any alteration. Nevertheless, it provides an awareness on how drugs can possibly influence age-at-death, sex, stature and ancestry or biological affinity estimation, amongst other traits such as pathological conditions. 

A number of questions arise from this review. One is that further research could target how medication may be affecting particular landmarks or refining those bone characteristics (e.g., location, dimensions, unilateral vs. bilateral, etc.), which may be used for biological reconstruction in forensic casework. Second, is that it may be worth seeing in terms of research what medical history a deceased had and explore whether this may have left any traits observed on their remains. Furthermore, it may be possible to consider medical history during our analysis of the individual, with regard to medication, in particular if forensic anthropologists have a medical background, or in conjunction with forensic pathologists and odontologists. As stable isotopes are also being used in forensic casework, a more in depth understanding of bone turnover may be worth exploring. 

Although these modifications on the skeleton are not uniquely specific to a given substance, they can suggest drug intake in the differential diagnosis. This is therefore an important piece information to be considered in forensic anthropological practice as it may implement the biological profile with unique information and improve accuracy in the application of standard methods and the interpretation of results. However, more research is needed to characterize with precision the effects of drugs on bones, and to clarify their influence on anthropological methods, for instance through the examination of skeletons with a known medical history of drug use. The knowledge reviewed in this study may be used in support or as basis for further research in forensic anthropology, but also potentially in the medical and pharmacological fields for/such as data to be shared more specifically.

## Figures and Tables

**Table 1:** Summary of the effects of drugs on bone, as taken from the literature review for this paper. For references or bibliography, see the main body of text.

Drug		Effect on Bone	Location
Cocaine		Cocaine-induced midline destructive lesion (CIMDL) and other nasal deformities, septum perforation, infection (e.g., maxillary sinusitis) Periodontitis, dental caries, (ante-mortem) tooth loss, dental erosion.	Nasal septum, nasal walls, hard palate, maxilla and orbital walls. Dentition.
Opioids	Morphine	Osteoporosis, osteopenia, increase risk of fracture, longitudinal growth, skeletal development.	Not specific. Some fractures may be at sites such as hip, spine, forearm but not always attributed to osteoporosis. Cartilage affected during growth and development.
	Methadone	Increased risk of osteoporosis and osteopenia, increased risk of fracture, decrease in bone mineral density.	Not specific. Some fractures may be at sites such as hip, spine, forearm but not always attributed to osteoporosis.
	Heroin	Decrease bone mineral density, osteoporosis, osteopenia, septic arthritis, bone turnover, osteomyelitis.	Not specific. Septic arthritis in sacroiliac, costoclavicular, hip and shoulder joint. Sometimes osteomyelitis in long bones at sites where injections.
Amphetamines		Osteonecrosis, Osteoporosis, Osteopenia, loss of bone density, maxillary sinusitis, osteomyelitis ‘Meth mouth’: Dental caries, periodontal disease, tooth loss, periodontitis, dental caries, dental erosion.	Loss of bone density throughout body. Osteonecrosis of jaw. Sinuses Dentition (‘Meth mouth’).
Cannabinoids		Possible loss of bone density, leading to osteoporosis and increased fracture risk. Periodontal disease.	Not specific. Dentition.
Alcohol		Effect on osteoblast proliferation, lower bone density, osteopenia, osteoporosis, increased fracture, poor fracture healing, avascular necrosis.	Throughout skeleton. This effect may depend on sex, age and lifestyle factors, patterns of drinking, volume of alcohol, etc. Avascular necrosis of femoral head.
Tobacco		Bone density, bone fractures, delayed haling of fractures or non-union. Periodontitis.	Throughout skeleton. Sites of osteoporotic fractures. No fractures to skull. Dentition.
Oral Glucocorticoids		Increased risk of osteoporosis, decrease in bone mineral density, fracture risk, slow fracture healing, delayed maturation, short stature.	Not specific.
Non-steroidal anti-inflammatory drugs (NSAIDs)		Delayed fracture and entheses healing. Fracture nonunion/malunion. Possible cartilage degeneration. Increase/decrease in BMD (type and dose-dependent). Possible increased/decreased fracture risk. Possible skeletal effects in fetus and newborn (therapy during pregnancy).	Changes not specific, observed hip, femur, spine, and forearm In fetus/newborn—cleft palate, fused ribs, decreased vertebral mineralization, deformation of lumbar arch, absent sacral arch, incomplete ossification of cervical arch, absent/hemicentric body of thoracic or lumbar vertebra.
Paracetamol		Possible decrease in BMD. Possible increased risk of fractures (at low doses). Possible impairment of implant osseointegration.	Observed in spine, hip, forearm.
Gonadotropin-releasing hormone (GnRH) agonist		Decrease in BMD (potentially reversible after treatment). Increased risk of fractures. Osteoporosis. Possible delay/attenuation of PBM.	Trabecular bone (lumbar spine), but also observed in hip, proximal femur, and radius.
Proton pump inhibitors		Increased risk of fractures. Osteoporosis. Possible decrease in BMD.	Any site, but in particular at the hips and lumbar vertebrae.
Antiretroviral therapy		Decrease in BMD, osteopenia, osteoporosis, osteonecrosis, osteomalacia, increased risk of fractures. Charcot joint (indirectly).	Throughout the skeleton, particularly at the femora, lumbar vertebrae and hips. Osteonecrosis on proximal femora, sometimes bilateral.
Antidepressant drugs		Reduced estrogen production. Osteoporosis. Increased risk of fracture. Decrease in bone mineral density.	Throughout skeleton. Osteoporotic fracture sites.
Anti-epileptic drugs		Decrease in bone mineral density and osteoporosis, increased risk of fracture, retarded growth and stunting.	Throughout skeleton.
Antidiabetic drugs		Decrease in bone mineral density, alteration of bone microstructure, increase risk of fractures, possibly osteoarthritis.	Throughout skeleton but increase risk in fracture particularly related to osteoporotic fracture sites.
Antiresorptive drugs		Osteonecrosis of the jaw.	In particular the mandible.
Antithrombotic drugs		Decrease in bone mineral density, increase risk of fractures and impaired fracture healing.	Throughout skeleton. Fractures at osteoporotic fracture sites.

**Table 2:** Summary of effects on bones according to the drug class discussed in this paper. The absence of any information in the cells does not necessarily mean that these changes do not occur in a particular drug, but it has not been noted in the literature consulted for this paper. Code: Y = yes.

	Decreased BMD/Osteoporosis	Increased Risk of Fractures	Bone Destruction/Osteonecrosis
Cocaine			Y
Methadone	Y	Y	Y
Heroin	Y	Y	Y
Amphetamines	Y		Y
Cannabinoids	Y?		
Alcohol	Y	Y	Y
Tobacco	Y	Y	
Oral glucocorticoids	Y	Y	
NSAIDs	Possibly Y (low doses)	Possibly Y (when BMD is decreased)	
Paracetamol	Possibly Y	Possibly Y	
GnRH agonist	Y	Y	
Proton pump inhibitors	Y	Y	
Antiretroviral therapy	Y	Y	Y
Antidepressant drugs	Y	Y	
Anti-epileptic drugs	Y	Y	
Antidiabetic drugs	Y	Y	
Antiresorptive drugs			Y
Antithrombotic drugs	Y	Y	

**Table 3:** Table that summarizes substances so far detected in bone through toxicological analyses in different studies. The table lists the substances, the study, the site of sampling, and the number of skeletons analyzed in the study.

Class of Molecules	Drugs	Bone Samples	Number of Individuals Analyzed	Reference
Amphetamines	MDA	Cranium	7	[27]
Anesthetics	Ketamine	Cranium, rib	19	[190]
Anticonvulsant drugs	Pregabalin	Rib	3	[189]
	Carbamazepine	Femur	36	[275]
Antidepressants	Amitriptyline	Iliac crest, vertebra	39	[22]
		Femur	36	[275]
		Femur	6	[23]
		Rib	7	[276]
	Citalopram	Cranium, rib	19	[190]
		Iliac crest, vertebra	39	[22]
	Dothiepin	Femur	36	[275]
	Doxepin	Femur	36	[275]
	Duloxetine	Rib	7	[276]
	Mianserin	Femur	36	[275]
	Moclobemide	Femur	36	[275]
	Nordoxepin (Metabolite of doxepin)	Femur	36	[275]
	Nortriptyline	Femur	36	[275]
	Trazodone	Cranium, rib	19	[190]
	Venlafaxine	Cranium, rib	19	[190]
		Rib	7	[276]
Antihistamine drugs	Diphenhydramine	Iliac crest, vertebra	39	[22]
Antihypertensive drugs	Atenolol	Rib	2	[277]
	Bisoprolol	Rib	2	[277]
Antipsychotics	Chlorpromazine	Femur	36	[275]
	Clozapine	Femur	36	[275]
	Haloperidol	Cranium, rib	19	[190]
	Mesoridazine	Femur	36	[275]
	Promazine	Cranium, rib	19	[190]
	Quetiapine	Cranium	19	[190]
		Rib	3	[189]
	Thioridazine	Femur	36	[275]
Benzodiazepines	Alprazolam	Cranium, rib	19	[190]
	Bromazepam	Femur	6	[23]
	Delorazepam	Vertebra, rib	7	[27]
		Cranium, rib	19	[190]
	Diazepam	Cranium vertebra, rib	7	[27]
		Cranium, rib	19	[190]
		Iliac crest, vertebra	39	[22]
		Femur	36	[275]
		Femur	6	[23]
	Flurazepam	Cranium, rib	19	[190]
	Lorazepam	Cranium	7	[27]
		Cranium, rib	19	[190]
	Lormetazepam	Cranium, rib	19	[190]
	Nordiazepam	Vertebra	7	[27]
		Cranium, rib	19	[190]
		Iliac crest, vertebra	39	[22]
		Femur	36	[275]
		Femur	6	[23]
	Oxazepam	Femur	36	[275]
	Temazepam	Femur	36	[275]
Cannabinoids	THCCOOH (Metabolite of THC)	Rib	7	[27]
Curare	Laudanosine (Metabolite of atracurium)	Iliac crest, vertebra	39	[22]
Opioids	6-MAM	Rib	6	[278]
	Buprenorphine	Vertebra	7	[27]
	Codeine	Iliac crest, vertebra	39	[22]
		Femur	36	[275]
		Femur	6	[23]
		Clavicle	3	[279]
	Meperidine	Iliac crest, vertebra	39	[22]
	Methadone	Cranium vertebra, rib	7	[27]
		Rib	6	[278]
		Femur	36	[275]
	Morphine	Rib	6	[278]
		Femur	6	[23]
		Femur	1	[280]
		Clavicle	3	[279]
	Norpropoxyphene (Metabolite of propoxyphene)	Iliac crest, vertebra	39	[22]
		Femur	36	[275]
	Oxycodone	Iliac crest, vertebra	39	[22]
		Femur	36	[275]
	Propoxyphene	Iliac crest, vertebra	39	[22]
		Femur	36	[275]
	Tramadol	Cranium, rib	19	[190]
		Rib	6	[278]
Stimulants	Caffeine	Femur	36	[275]
	Cocaine	Cranium, rib	19	[190]
		Femur	6	[23]
	Benzoylecgonine (Metabolite of cocaine)	Vertebra, rib	7	[27]
		Cranium, rib	19	[190]
		Rib	6	[278]
		Iliac crest, vertebra	39	[22]
		Femur	6	[23]

**Table 4:** Awareness of how drugs could affect biological profile reconstruction in forensic anthropology.

	Possible Effects	Observations
Age-at-death	Delayed maturation, pre-mature (costal) cartilage calcification, pubic symphysis morphology, joint disease, osteoporosis, tooth loss.	Likely age overestimation in adults. May require imaging such as body CT scans. Moreover, similar effects when estimation the age of a living person. If anomalies in age indicators perhaps enquire re medication and lifestyle environment.
Sex estimation	Possible morphological changes in pelvis and skull.	Misdiagnosis. Research in transgender individuals required too.
Physical attributes (stature, ancestry or population affinity)	Morphological assessment of nasal area may be altered by drug abuse. Stunted growth.	Ancestry estimation, stature.
Unique features	Osteonecrosis of the jaw, dental problems, fracture patterns.	May be able to indicate some possible medications or be consistent with medication intake.
